# Alleviating dormancy in *Brassica oleracea* seeds using NO and KAR1 with ethylene biosynthetic pathway, ROS and antioxidant enzymes modifications

**DOI:** 10.1186/s12870-019-2118-y

**Published:** 2019-12-23

**Authors:** Abdul Sami, Muhammad Waheed Riaz, Xiangyu Zhou, Zonghe Zhu, Kejin Zhou

**Affiliations:** College of Agronomy, Anhui Agricultural University, Hefei, 230036 China

**Keywords:** *Brassica oleracea*, KAR1, Nitric oxide, Ethylene, Seed dormancy, Seed germination, ROS, Antioxidant enzymes

## Abstract

**Background:**

Seed dormancy is a prevailing condition in which seeds are unable to germinate, even under favorable environmental conditions. Harvested *Brassica oleracea* (Chinese cabbage) seeds are dormant and normally germinate (poorly) at 21 °C. This study investigated the connections between ethylene, nitric oxide (NO), and karrikin 1 (KAR1) in the dormancy release of secondary dormant *Brassica oleracea* seeds.

**Results:**

NO and KAR1 were found to induce seed germination, and stimulated the production of ethylene and 1-aminocyclopropane-1-carboxylic acid (ACC), and both ethylene biosynthesis enzyme ACC oxidase (ACO) [1] and ACC synthase (ACS) [2]. In the presence of NO and KAR1, ACS and ACO activity reached maximum levels after 36 and 48 h, respectively. The inhibitor of ethylene 2,5-norbornadiene (NBD) had an adverse effect on *Brassica oleracea* seed germination (inhibiting nearly 50% of germination) in the presence of NO and KAR1. The benefits from NO and KAR1 in the germination of secondary dormant *Brassica oleracea* seeds were also associated with a marked increase in reactive oxygen species (ROS) (H_2_O_2_ and O_2_˙ˉ) and antioxidant enzyme activity at early germination stages. Catalase (CAT) and glutathione reductase (GR) activity increased 2 d and 4 d, respectively, after treatment, while no significant changes were observed in superoxide dismutase (SOD) activity under NO and KAR1 applications. An increase in H_2_O_2_ and O_2_˙ˉ levels were observed during the entire incubation period, which increasing ethylene production in the presence of NO and KAR1. Abscisic acid (ABA) contents decreased and glutathione reductase (GA) contents increased in the presence of NO and KAR1. Gene expression studies were carried out with seven ethylene biosynthesis ACC synthases (ACS) genes, two ethylene receptors (ETR) genes and one ACO gene. Our results provide more evidence for the involvement of ethylene in inducing seed germination in the presence of NO and KAR1. Three out of seven ethylene biosynthesis genes (*BOACS7, BOACS9* and *BOACS11*), two ethylene receptors (*BOETR1* and *BOETR2*) and one ACO gene (*BOACO1*) were up-regulated in the presence of NO and KAR1.

**Conclusion:**

Consequently, ACS activity, ACO activity and the expression of different ethylene related genes increased, modified the ROS level, antioxidant enzyme activity, and ethylene biosynthesis pathway and successfully removed (nearly 98%) of the seed dormancy of secondary dormant *Brassica olereace* seeds after 7 days of NO and KAR1 application.

## Background

Seed dormancy is a prevailing condition in which seeds are unable to germinate even in a favorable environment [1]. Seed dormancy can be divided into two types: [1] primary dormancy and [2] secondary dormancy. Primary dormancy is caused by the interaction between environmental factors and abscisic acid (ABA) in the late stages of seed development, while secondary dormancy is induced at different development stages and is caused by abiotic stresses including anoxia, temperature, and light [2]. Oxygen deficiency can increase the intensity of secondary dormancy [2]. Many phytohormones such as cytokinins, brassinosteroids, nitric oxide, and ethylene can play crucial roles in the alleviation of seed dormancy and germination [3, 4]. The germination of dormant seeds of different plant species can also be regulated by a plant-derived smoke compound called karrikin-1 (KAR1) or butenolide [5]. It has been hypothesized that karrikins are a family of different isoforms. Six isoforms (KAR1 to KAR6) in the KARs group and others in smoke [6] have been identified, and these endogenous compounds are considered as the plant’s hormones [7].

NO is a signaling molecule that associates with plant hormones to break seed dormancy [8]. During seed germination, the post-translational modifications of NO-dependent proteins are key mechanisms [9]. Acidified KNO_2_, *S-nitroso-N-acetylpenicillamine* (SNAP), and *S-nitrosoglutathione* (GSNO) are the main donors of NO that help to release the dormancy conditions of seeds in different plant species, such as lettuce, redroot pigweed, barleyand apple [10–13]. Previous studies have shown that apple seed dormancy release can be achieved in the presence of the interaction between nitric oxide and ethylene biosynthesis [8, 13, 14].

The phytohormone known as ethylene is involved in many important mechanisms in plants, such as ripening and reacting to various stresses [15–17]. It also has a pivotal role in the control of early germination and the alleviation of seed dormancy [18]. S-adenosyl-methionine produces ethylene in seeds in two steps: [1] conversion of *1-aminocyclopropane-1-carboxylic acid* (ACC) in the presence of ACC synthase (ACS); and [2] the oxidation of ACC [17, 19], which is the same pathway that was discovered from other plant parts. ACC synthase is considered an essential enzyme for ethylene production, while ACO is an enzyme that controls ethylene evolution to break seed dormancy [20]. In different plant species, ACS and ACO are two families and both contain multiple genes [21]. The members of these families regulate the transition from seed dormancy to non-dormancy [22]. Under cold stress, all *AtACO* genes and two ACS genes (*AtACS2* and *AtACS11*) were down-regulated in *A. thaliana* [23–25], and *FsACO1* transcript levels had been up-regulated to break seed dormancy in *Fagus sylvatica* [26]. In wheat grains, the expressions level of some *TaACOs* genes increase after ripening [27].

Reactive oxygen species (ROS) and reactive nitrogen species, such as hydroxyl radical (^•^OH), hydrogen peroxide (H_2_O_2_), superoxide anion (O_2_˙ˉ), hydroxylamines, and nitrates are involved in the alleviation of seed dormancy in a number of plant species [28–30]. Seed germination usually starts when the level of reactive nitrogen species or reactive oxygen species reaches a certain level [31]. ROS accumulation releases sunflower seed dormancy [32]. Appropriate levels of ROS strongly correlate with the concentration and enzymatic activity of its compounds, such as catalase (CAT), glutathione reductase (GR), superoxide dismutase (SOD), mono-de-hydro-ascorbate reductase (MDHAR), and peroxidases, as well as non-enzymatic compound such as ascorbate [29–32]. In monocotyledonous and dicotyledonous species, H_2_O_2_ has been considered the essential ROS in releasing seed dormancy [33]. Superoxide is converted into hydrogen peroxide in the presence of SOD after the conversion CAT starts its process and breaks H_2_O_2_ into oxygen and water, and these two enzymatic antioxidants SOD and CAT also cooperate with the ascorbate-glutathione cycle, dehydroascorbate reductase (DHAR), ascorbate peroxidase (APX) and glutathione reductase (GR) [34, 35]. Earlier studies explored that, sensitivity and balance between GA and ABA are causes of seed dormancy regulation [36]. The ABA is a key hormone in seeds and it is also responsible to maintain the seed dormancy, so decrease in ABA level reduces the seed dormancy [37]. GA is also considered as important hormone and helpful to release seed dormancy. In number of plant, GA can regulate seed germination and decreases dormancy while ABA regulates seed dormancy [38].

Previous data have shown that after harvest, *Brassica oleracea* (Chinese cabbage) seeds exhibit dormancy during a certain period of time [39]. *Brassica* species have no or little primary dormancy because primary dormancy usually establish in wild plants [39]. However, under environmental stress, secondary dormancy can be induced in *Brassica* cultivars [39]. In rapeseed, some abiotic factors such as changes in temperature, light intensity, humidity, and air pressure can have an effect on seed dormancy and germination [40]. Successful breakage of *Brassica* seed dormancy has been recorded under particular conditions such as light/dark regimes; alternating day/night temperatures; and HCN, NaClO, NO and ethylene interactions [41–43]. Therefore the aim of this study was to examine whether germination induction of dormant *Brassica oleracea* seeds by NO and KAR1 is associated with ethylene modification, control of ROS (H_2_O_2_ and O_2_**˙ˉ)**, and the enzymatic antioxidants activity.

## Results

### Effects of NO and KAR1 on germination

**Fig. 1:**
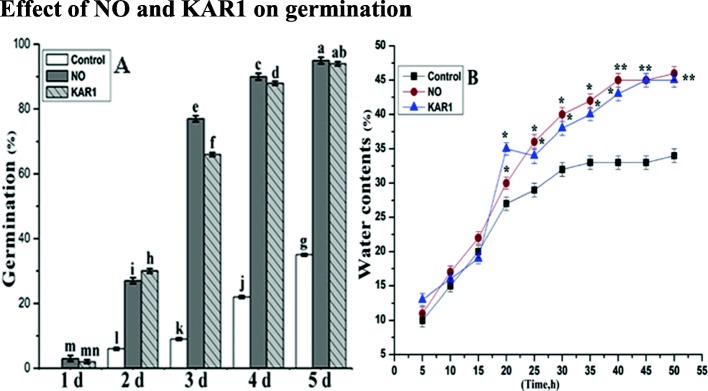
Effects of NO (5 mM) and KAR1 (3 × 10^− 9^ M) on (**a**) Seed germination rate, **b** water contents in secondary dormant *Brassica oleracea* seeds during imbibition at 21 °C (*n* = 3). Values are mean ± SE. Significant differences between treatments determined by two-way analysis of variance (ANOVA), followed by HSD Tukey’s test. Means with different letters are significantly different at *p < 0.05* and *P* ≤ 0.01. KAR1, karrikin1; NO, Nitric oxide; h, hours

NO (5 mM) and KAR1 (3 × 10^− 9^ M) are very effective in breaking seed dormancy and increasing seed germination. Secondary dormant *Brassica oleracea* L. seeds were treated with NO (5 mM) and KAR1 (3 × 10^− 9^ M). Under favorable conditions, non-treated secondary dormant *Brassica oleracea* seeds were unable to germinate, and after 3 days of sowing only 9% germination was noted at 21 °C, as shown in Fig. 1a. However, under nitric oxide and KAR1 treatments, the germination rate was enhanced after 48 h. The germination rate increased even more after 4 and 5 days of nitric oxide and KAR1 applications. The germination rate actually correlated quite well with the treatment time; nearly all seeds were able to germinate as the treatment time increased. We observed that the effects of nitric oxide and karrikin1 were dependent on the duration of treatment.

In order to check the efficiency of NO and KAR1 on water uptake ability, secondary dormant *Brassica oleracea* seeds were incubated in water. The water contents of NO and KAR1-treated seeds were recorded at 5, 10, 20, 30, 40 and 50 h. *Brassica oleracea* seeds treated with NO or KAR1 showed a rapid increase in water uptake, reaching 28–35% after the initial 20 h of incubation (Fig. 1b). After 30 h of KAR1 and NO treatments, 38–40% water uptake was observed. Furthermore, after 40 h of imbibition in KAR1 and NO, water uptake increased to about 42–45% and continued to increase slightly until the end of incubation at 50 h.

### Hydrogen peroxide and superoxide anion accumulation

**Fig. 2:**
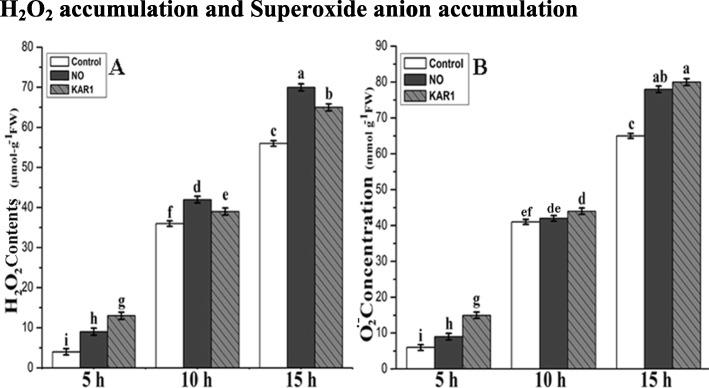
Effects of NO (5 mM) and KAR1 (3 × 10^− 9^ M) on (**a**) H_2_O_2_, **b** O_2_^-•^ in secondary dormant *Brassica oleracea* seeds during imbibition at 21 °C (*n* = 3). Values are mean ± SE. Significant differences between treatments determined by two-way analysis of variance (ANOVA), followed by HSD Tukey’s test. Means with different letters are significantly different at *p < 0.05*. KAR1, karrikin1; NO, Nitric oxide; H_2_O_2,_ hydrogen peroxide; O_2_^•-^ Superoxide anion, h, hours

As mentioned above, the increase in reactive oxygen species and enzymatic activities to certain levels can alleviate seed dormancy. Because of these results, we also measured H_2_O_2_ and superoxide anion concentrations in treated and non-treated samples. H_2_O_2_ concentration was measured after 1, 2, 3 and 4 d of treatments with NO (5 mM) and KAR1 (3 × 10^− 9^ M). A rapid increase in H_2_O_2_ concentration was observed during NO and KAR1 treatment after 1, 2, 3 and 4 d (Fig. 2a), and the level of H_2_O_2_ was lower in control samples than in treated. H_2_O_2_ concentration was 6.5 μmol g^−1^FW in control samples after 1 d of imbibition in water, and increased more than twofold under NO or KAR1 treatments. H_2_O_2_ concentration after 1 day was recorded as 6.5 (μmol-g^−1^FW) in control samples, 15 (μmol-g^−1^FW) in NO, and 17 (μmol-g^−1^FW) in KAR1 treated samples. H_2_O_2_ concentration after 2 d rapidly increased to 57 (μmol-g^−1^FW) and 46 (μmol-g^−1^FW) under NO and KAR1 treatments, respectively. After 3 d of treatment, concentration was 65 (μmol-g^−1^FW) in control samples, 80 (μmol-g^−1^FW) in NO treated samples and 78 (μmol-g^−1^FW) in KAR1 treated samples. The maximum H_2_O_2_ concentration was noted at the end of 4 d under NO and KAR1 treatments. After 4 d of treatment, concentration was 74 (μmol-g^−1^FW) in control samples, 85 (μmol-g^−1^FW) in NO treated samples and 82 (μmol-g^−1^FW) in KAR1 treated samples.

Superoxide anion concentration was lower in non-treated samples after 1, 2, 3 and 4 d of imbibition in water. In the presence of KAR1, we recorded a considerable increase in superoxide accumulation, while samples treated with NO had a lower stimulatory effect than KAR1 after 1 d (Fig. 2b). The superoxide anion level increased rapidly after 2, 3, and 4 d of incubation in NO or KAR1 solutions (Fig. 2b). The superoxide anion level in control samples after 1 d was 16 (μmol-g^−1^FW), 19 (μmol-g^−1^FW) in NO samples, and 18 (μmol-g^−1^FW) in KAR1 treated samples. The superoxide anion level after 2 d increased up to 51 (μmol-g^−1^FW) in control samples, 55 (μmol-g^−1^FW) under NO, and 58 (μmol-g^−1^FW) under KAR1 treatments. Moreover, after 3 d of treatments, the superoxide anion level was recorded as 75 (μmol-g^−1^FW) in control samples, 81 (μmol-g^−1^FW) in NO, and 84 (μmol-g^−1^FW) in KAR1 treated samples. After 4 d of treatments, the superoxide anion level had increased to 79 (μmol-g^−1^FW) in control samples, 86 (μmol-g^−1^FW) in NO, and 88 (μmol-g^− 1^FW) in KAR1 treated samples.

### The activities of SOD, CAT and GR

**Fig. 3:**
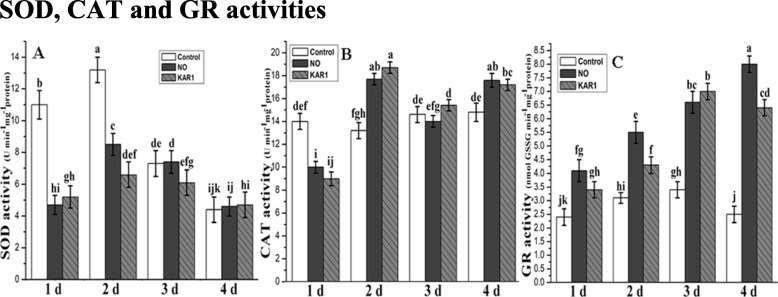
Effects of NO (5 mM) and KAR1 (3 × 10^− 9^ M) on (**a**) SOD, **b** CAT, **c** GR in secondary dormant *Brassica oleracea* seeds during imbibition at 21 °C (*n* = 3). Values are mean ± SE. Significant differences between treatments determined by two-way analysis of variance (ANOVA), followed by HSD Tukey’s test. Means with different letters are significantly different at *p < 0.05*. KAR1, karrikin1; NO, Nitric oxide; SOD, Superoxide dismutase; CAT, Catalase; GR, Glutathione reductase; d, days

An imbalance among enzymatic activities can also cause seed dormancy. Secondary dormant *Brassica oleracea* seeds were treated with NO (5 mM) and KAR1 (3 × 10^− 9^ M) for 1, 2, 3 and 4 d, and SOD, CAT and GR activities were measured. The highest SOD activity was recorded in control *Brassica oleracea* embryos after 1 d (11 U min^− 1^ mg^− 1^ proteins) and 2 d (13 U min^− 1^ mg^− 1^ proteins), and2–3 times was higher and significantly different than treated samples (Fig. 3a). These results confirm the differences in SOD activity between treated and non-treated embryos, and how the SOD level gradually reduced after 3 and 4 d. No significant difference was observed between control, NO and KAR1 treatments after 4 d of treatments. Overall, SOD activity decreased as the time of treatments increased. SOD activity was lower in samples under NO or KAR1 treatments than the control samples after 1, 2, 3 and 4 d. In addition, SOD activity after 2 d of NO application was comparatively higher than KAR1 treated samples.

The catalase activity in *Brassica oleracea* embryos during the whole experiment stayed on a steady level in control samples; however, after 2 d of imbibition in water, the values decreased (Fig. 3b). NO and KAR1-treated embryos exhibited a slight increase in CAT activity after 2 and 4 d. Under KAR1 treatment, CAT activity was at a maximum (19 U min^− 1^ mg^− 1^ proteins) after 2 d; under NO treatment, the maximum value was 18 (U min^− 1^ mg^− 1^ proteins) after 4 d of treatment; and in control samples, the maximum CAT value (15 U min^− 1^ mg^− 1^ proteins) was recorded after 3 d.

The lowest GR activity was recorded in the control samples. The GR activity increased to 3.3 (nmol GSSG min^− 1^ mg^− 1^ proteins) in control samples after 2 and 3 d, but after 4 d, the GR activity went back to its initial rate (Fig. 3c). Meanwhile, in treated embryos, the GR activity increased continuously during germination with NO treatment, and GR values increased about three times and were significantly different than the control embryos after 4 days of imbibition (Fig. 3c). The maximum GR value 8 (mol GSSG min^− 1^ mg^− 1^ proteins) was recorded after 4 d under NO treatment; under KAR1 treatment, the maximum value 7 (mol GSSG min^− 1^ mg^− 1^ proteins) was measured after 3 d of treatment.

### The effect of KAR1 and NO on the contents of ABA and GA

**Fig. 4:**
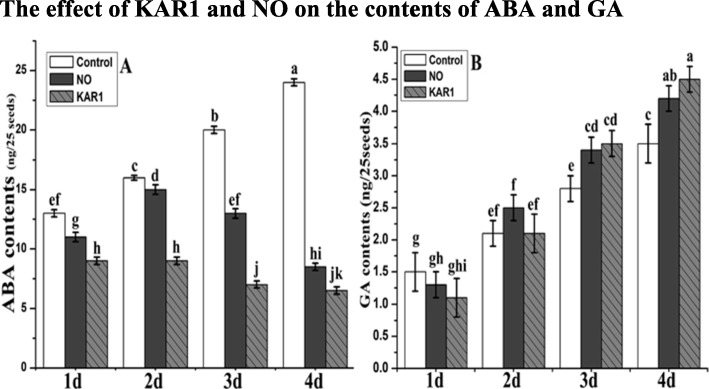
Effects of NO (5 mM) and KAR1 (3 × 10^− 9^ M) on (**a**) ABA contents, **b** GA contents in secondary dormant *Brassica oleracea* seeds during imbibition at 21 °C (*n* = 3). Values are mean ± SE. Significant differences between treatments determined by two-way analysis of variance (ANOVA), followed by HSD Tukey’s test. Means with different letters are significantly different at *p < 0.05*. KAR1, karrikin1; NO, Nitric oxide; ABA, abscisic acid; GA, gibberellic acid; d, days

Balance between ABA and GA are responsible for the regulation of the dormancy state and germination. NO (5 mM) and KAR1 (3 × 10^− 9^ M) are very helpful to remove seed dormancy and increase seed germination. To check this we measured the ABA and GA contents in the presence of NO (5 mM) and KAR1 (3 × 10^− 9^ M) for 1, 2, 3 and 4 d (Fig. 4a and b). We recoded the increasing pattern of ABA and GA contents in control samples during whole incubation period. Highest ABA (24 ng/25 seeds) and GA contents (5.5 ng/ 25 seeds) were recorded after 4 d of application in non-treated samples (Fig. 4a and b). Lowest ABA contents (6.5 ng/25 seeds) were recorded after 4 d of KAR1 application. Increase in GA contents was noticed during the whole incubation period. After 4 d of incubation higher GA contents were recorded in the presence of KAR1 (Fig. 4b).

### Ethylene production and ethylene inhibitor NBD

**Fig. 5:**
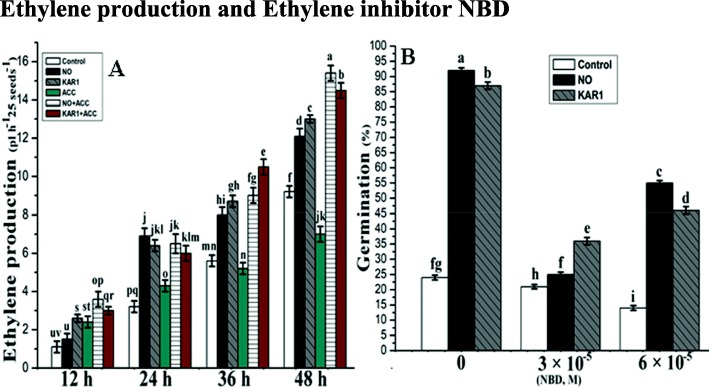
Effects of NO (5 mM) and KAR1 (3 × 10^− 9^ M) on (**a**) Ethylene Production, **b** NBD in secondary dormant *Brassica oleracea* seeds during imbibition at 21 °C (*n* = 3). Values are mean ± SE. Significant differences between treatments determined by two-way analysis of variance (ANOVA), followed by HSD Tukey’s test. Means with different letters are significantly different at *p < 0.05*. KAR1, karrikin1; NO, Nitric oxide; NBD_,_ 2,5-norbornadiene; h, hours

An increase in ethylene production is known to play a major role in alleviating seed dormancy. To verify this, we measured ethylene production in the presence of ethylene inhibitor NBD and the increase/decrease in ethylene production in the presence of NO (5 mM) and KAR1 (3 × 10^− 9^ M). Ethylene production rates, either in water or NO and KAR1-treated, were determined at different time-points (12, 24, 36 and 48 h) of imbibition (Fig. 5a). Seeds were incubated in NO and KAR1 or in water for 12, 24, 36, and 48 h. ACC (1 × 10^− 4^ M) was also applied, either alone or in combination with NO or KAR1. The level of ethylene was lower at 12 h of incubation in the presence of NO. The untreated seeds started producing ethylene after 24 h of incubation at a relatively low stable level. However, in the presence of ACC, no significant changes were observed in ethylene production. Both NO and KAR1 applied in combination with ACC increased ethylene production about twofold after 48 h of imbibition, indicating the ACO activation.

An ethylene binding inhibitor, NBD (6 × 10^− 5^ and 3 × 10^− 5^ M) was applied to also examine whether ethylene action is involved or not in the response of secondary dormant seeds to NO and KAR1. *Brassica oleracea* seeds germinated poorly in control samples at 21 °C after 4 d (Fig. 5b), while seeds treated with NO (5 mM) and KAR1 (3 × 10^− 9^ M) showed 92% germination. Our experiment also demonstrated that ethylene signaling inhibitors like NBD can markedly inhibit the germination of *Brassica* seeds induced by NO and KAR1. Only 25–55% of NO seed germinations and 36–46% of KAR1 seed germinations were recorded, as shown in (Fig. 5b).

### ACC contents and activities of ACS and ACO

**Fig. 6:**
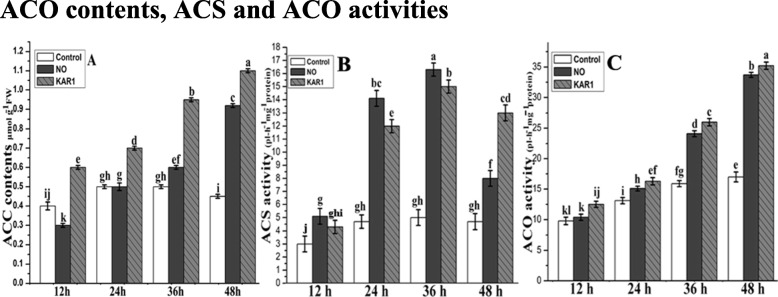
Effects of NO (5 mM) and KAR1 (3 × 10−9 M) on (**a**) ACC contents, (**b**) ACS activity, (**c**) ACO activity in secondary dormant *Brassica oleracea* seeds during imbibition at 21 °C (*n* = 3). Values are mean ± SE. Significant differences between treatments determined by two-way analysis of variance (ANOVA), followed by HSD Tukey’s test. Means with different letters are significantly different at *p < 0.05*. KAR1, karrikin1; NO, Nitric oxide; ACC synthase, ACS, ACC oxidase, ACO; h, hours

ACC is the direct precursor of ethylene biosynthesis. In order to determine ACC contents, seeds were incubated in NO (5 mM) and KAR1 (3 × 10^− 9^ M) solutions or in water for 12, 24, 36, and 48 h. ACC contents recorded at 12 and 48 h of incubation in water were 0.4 and 0.45 μmol g^−1^FW, respectively. Little increase (0.5 μmol g^−1^FW) was recorded after that at 24 and 36 h, while little decrease (0.45 μmol g^−1^FW) was noted after 48 h (Fig. 6a). In the presence of NO at 12 h, ACC contents were low (0.3 μmol g^−1^FW), but started increasing over time and maximum ACC contents (0.92 μmol g^−1^FW) were noted after 48 h (Fig. 6a). The same pattern was observed in the presence of KAR1: after 12 h, ACC contents were 0.6 μmol g^−1^FW (twice that of the NO treatment), and a continuous increase was observed in the presence of KAR1 until the end of incubation. After 48 h, maximum ACC contents (1.1 μmol g^−1^FW) were noted (Fig. 6a).

ACS and ACO activities increase ethylene production and are (directly or indirectly) helpful in alleviating seed dormancy. To determine ACS and ACO activities, seeds were incubated in water or NO (5 mM) and KAR1 (3 × 10^− 9^ M) solutions for 12, 24, 36, and 48 h. The ACS activity was relatively low with no significant changes observed in the control culture (Fig. 6b), while the ACS activity increased two to three-fold after 24 and 36 h of incubation in the presence of NO and KAR1. However, in NO pretreated samples, the ACS level decreased after 48 h of imbibition.

ACO activity was also low at the start of the incubation period, in both treated and non-treated samples (Fig. 6c). However, the ACO activity increased rapidly after 36 h of both NO and KAR1 treatments, a twofold increase in comparison to the control (Fig. 6c). The significant stimulatory effects of NO and KAR1 on ACO activity in vitro were also observed after 48 h of imbibition (Fig. 6c), and ACO activity reached its maximum.

### Relative expression of *Brassica oleracea* ethylene-related genes

**Fig. 7:**
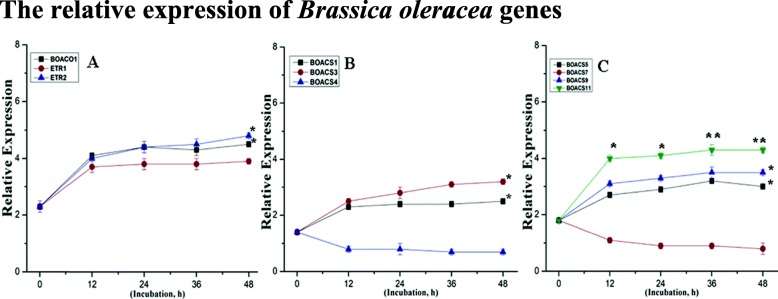
Relative expression patterns of BOACO1 and ethylene receptor genes (**a**) and ethylene biosynthesis genes (BOACS1, BOACS3, BOACS4, BOACS5, BOACS7, BOACS9, and BOACS11) (**b**, **c**) in secondary dormant *Brassica oleracea* seeds incubated in water at 21 °C. Transcript levels for each gene were estimated by qRT-PCR. The fold changes indicate the expression patterns of analyzed genes relative to their transcript levels in dry seeds (0 h of incubation) with assumed value of 1. Statistical analyses: two-way ANOVA with post hoc Tukey’s (HSD) test with confidence interval 0.05, significance between groups indicated as * for *P* ≤ 0.05 and ** for *P* ≤ 0.01

To thoroughly check the effects of NO (5 mM) and KAR1 (3 × 10^− 9^ M) on ethylene production, we observed the expression of ethylene-related genes. The relative expression of genes encoding biosynthetic enzymes (one ACO oxidase (*BOACO1*), seven ACS synthases (*BOACS1, BOACS3, BOACS4, BOACS5, BOACS7, BOACS9*, and *BOACS11*) and two ethylene receptor (*BOETR1* and *BOETR2*) genes) was analyzed during the imbibition of seeds in water at different incubation periods (0, 12, 24, 36 and 48 h) (Fig. 7a, b and c). When compared to dry (0 h) seeds, the level of *BOACS1* transcripts remained unchanged during the whole period of incubation, while *BOACS3, BOACS5, BOACS9* and *BOACS11* were up-regulated. However *BOACS4* and *BOACS7* were down-regulated to a different extent during incubation in water (Fig. 7a, b and c). Both *BOETR1* and *BOETR2* were also up-regulated, as shown in Fig. 7a.

**Fig. 8:**
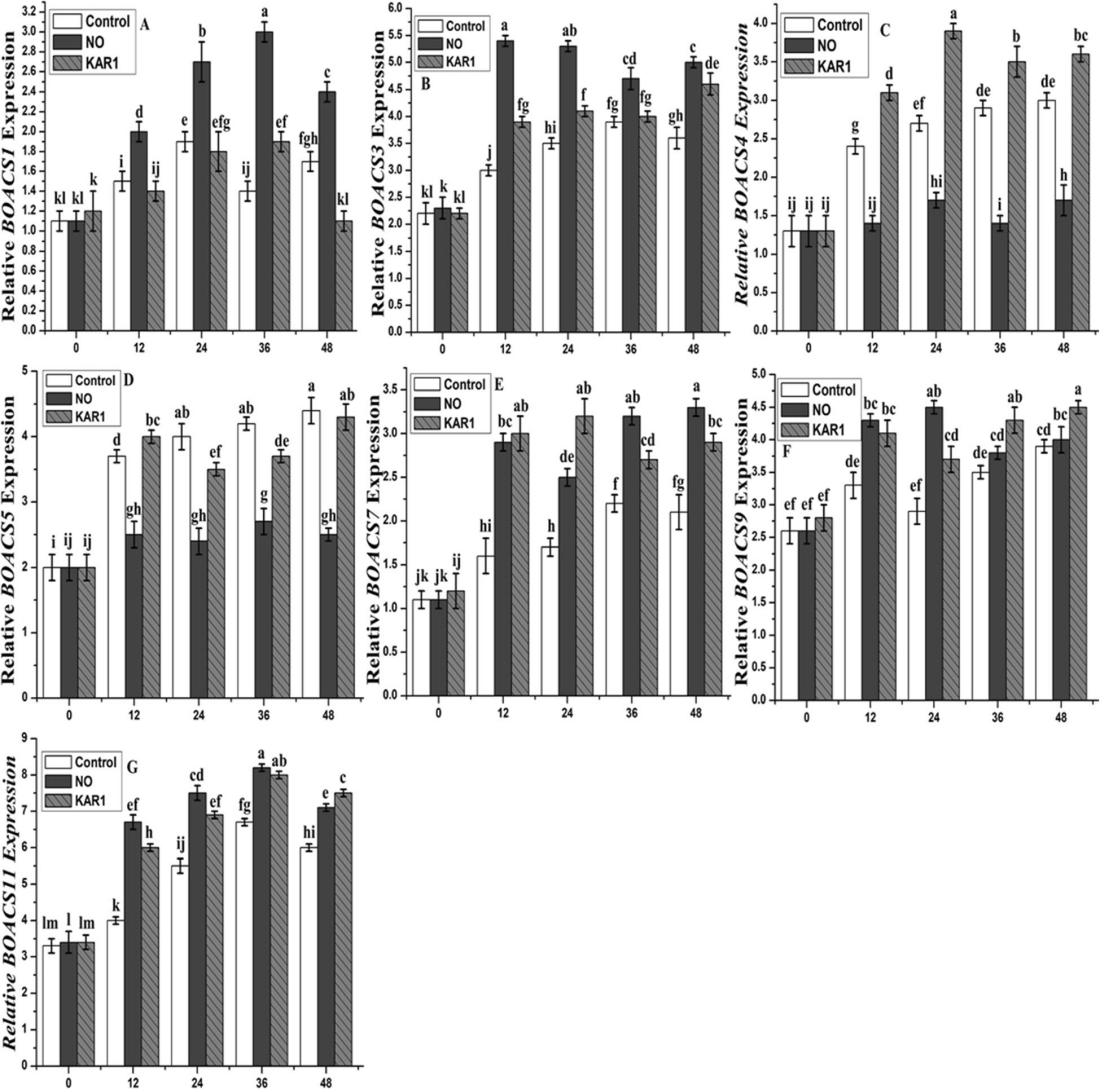
Effects of NO (5 mM) and KAR1 (3 × 10^− 9^ M) on (**a**) *BOACS1*, **b** *BOACS3,* **c** *BOACS4,* **d** *BOACS5,* **e** *BOACS7,* **f** *BOACS9*, **g** *BOACS11* genes in secondary dormant *Brassica oleracea* seeds during imbibition at 21 °C (*n* = 3). Values are mean ± SE. Significant differences between treatments determined by two-way analysis of variance (ANOVA), followed by HSD Tukey’s test. Means with different letters are significantly different at *p < 0.05*. KAR1, karrikin1; NO, Nitric oxide; h, hours

**Fig. 9:**
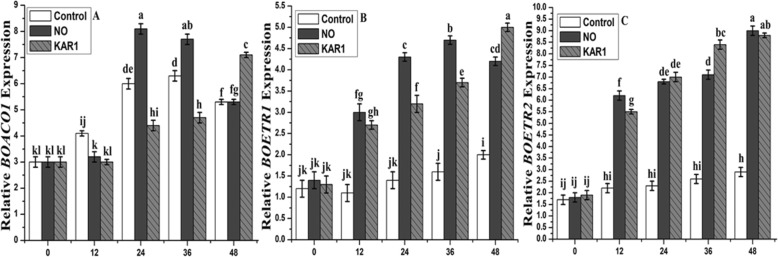
Effects of NO (5 mM) and KAR1 (3 × 10^− 9^ M) on (**a**) *BOACO1*, **b** *BOETR1,* **c** *BOETR2* genes in secondary dormant *Brassica oleracea* seeds during imbibition at 21 °C (*n* = 3). Values are mean ± SE. Significant differences between treatments determined by two-way analysis of variance (ANOVA), followed by HSD Tukey’s test. Means with different letters are significantly different at *p < 0.05*. KAR1, karrikin1; NO, Nitric oxide; h, hours

In the presence of NO (5 mM) and KAR1 (3 × 10^− 9^ M), gene expression analyses were also carried out for all genes mentioned above with different incubation periods (0, 12, 24, 36 and 48 h) (Fig. 8). *BOACT1* (actin gene) was used as the reference gene. Almost no change was observed in the expression of all genes at 0 h (Fig. 8). The level of *BOACS1* was lower after 12, 36 and 48 h, but after 24 h, the level was higher in the presence of KAR1 (3 × 10^− 9^ M) than the control treatment (Fig. 8a and b). In the presence of KAR1, the expression of *BOACS2* was higher after 12, 24 and 48, but was nearly the same after 36 h in comparison to the control treatment. While in the presence of NO (5 mM), the levels of both genes were higher and a significant difference was noted in comparison to the control. *BOACS4* and *BOACS5* showed lower expression under NO application in comparison to KAR1 and control (Fig. 8c and d). The expression levels of the remaining three genes (*BOACS7, BOACS9* and *BOACS 11*) were higher and more significant in both treatments than in the control treatment (Fig. 8e, f and g). The ethylene receptor genes, *BOETR1* and *BOETR2,* were up-regulated to a different extent during imbibition (Fig. 9b and c). *BOETR1* showed a stable twofold induction of its transcriptional activity when measured 12 to 48 h after imbibition (Fig. 9b), while about a threefold change in the transcript level was observed for *BOETR2* (Fig. 9c). Contrasting results were obtained for genes encoding biosynthetic enzymes (Fig. 9a). The expression of the *BOACO1* gene was higher after 36 and 48 h of incubation (Fig. 9a) in the presence of NO, while the presence of KAR1 showed a slightly lower expression level than the control.

## Discussion

### Effect of NO and KAR1 on seed germination, hydrogen peroxide and superoxide anion levels

Our experimental data showed that secondary dormant *Brassica oleracea* seeds (poorly germinated at 21 °C) are in a state of dormancy (Fig. 1). The NO (5 mM) and KAR1 (3 × 10^− 9^ M) treatments effected on *Brassica oleracea* dormant seeds germination (Fig. 1a). NO and KAR1 treatments successfully removed the seed dormancy in secondary dormant *Brassica oleracea* seeds. Our findings (Fig. 1) that both NO and KAR1 can break seed dormancy and induce seed germination is in agreement with previous studies [13, 44]. Past studies have also proved that the NO present in the surrounding environment can enhance germination at the early stages of seed germination [45]. However, further investigations are still required to fully understand the regulatory role of NO and KAR1 in breaking dormancy.

Seed dormancy alleviation not only depends on plant hormones, but it is also regulated by ROS (H_2_O_2_ and (O_2_˙ˉ) activities. ROS are small molecules in plant tissues and seeds that play a dual role in toxicity and signaling [31]. In the seed germination process, ROS are released into the surrounding environment to facilitate radical production [46]. Previous studies have shown that ROS accumulation is essential for various plant species in breaking seed dormancy and regulating seed germination [31, 47]. KAR1 and NO-induced seed germination is also associated with H_2_O_2_ and O_2_˙ˉ accumulation (Fig. 2). In the early stages of seed germination, the production of ROS (H_2_O_2_ and O_2_˙ˉ) indicates a positive biological reaction with successive germination and vigorous seedling growth. A previous study also confirmed that H_2_O_2_ production can break seed dormancy during the early phases of seed germination [32]. The data in our experiment show that H_2_O_2_ and O_2_˙ˉ accumulation/application were not harmful to *Brassica* seeds with a high viability (expressed as seed germination) in NO and KAR1 treated samples. This may be due to the cellular antioxidant system [48], which is also implied in Fig. 2. Similar interesting results were also noticed in apple seeds treated with NO and HCN [11].

### Effect of NO and KAR1 on antioxidant enzymes, ABA and GA level

The stimulatory effect of KAR1 on seed dormancy and seed germination has been reported by many researchers [11, 44, 49]. SOD is the primary antioxidant [50], playing a crucial role in controlling plant oxidative stress. SOD and CAT are mainly localized in mitochondria and peroxisomes, and are not sensitive to H_2_O_2_. Our study indicates that *Brassica oleracea* seeds treated with NO and KAR1 did not significantly change CAT and SOD activity only a slight stimulation of CAT activity (Fig. 3) was recorded. Additionally, neither NO nor KAR1 significantly altered SOD and CAT activity (Fig. 3). NO and KAR1 enhanced CAT and SOD activities to their maximum level after 2 days of treatments. Subsequently, SOD levels decreased to 5% as the time of treatment increased (Fig. 3). CAT activity also decreased after 3 and 4 days of application (Fig. 3). Similar to our findings, previous studies have demonstrated that gibberellic acid down-regulates SOD and CAT activities in barley [51], and in *A. fatua,* CAT and SOD activity were down-regulated by GA3 and KAR1 [52]. NO and KAR1 progressively enhance the GR level (Fig. 3c). Comparison of the GR activity and CAT and SOD activity in NO- and KAR1-treated *Brassica* samples suggest that GR activity is positively correlated with duration of application.

In the present study we observed the combined effect of KAR1 and NO on ABA and GA to release the seed dormancy (Fig. 4). Both NO and KAR1 were found to reduce ABA contents and increase in GA contents. In previous studies, GA is observed as negative while ABA is noted as positive regulator for seed dormancy development [18, 53]. Dormancy release by KAR1 and NO in *Brassica* was accompanied by degradation of ABA level (Fig. 4). Previous [38] study also supported our results; seed dormancy release also depends upon an increase in GA contents and decrease in ABA contents. Previous studies exhibited that in dormant *Arabidopsis thaliana* seeds, KAR1 is required for GA biosynthesis [6].

### Effect of NO and KAR1 on ethylene and ethylene inhibitor NBD

The role of ethylene in the alleviation of seed dormancy and the regulation of seed germination is still not fully understood [20]. Our study, combined with an analysis of the ethylene biosynthetic pathway and application of NBD, clearly show that in the presence of NBD, NO and KAR1 have no significant effect on seed germination (Fig. 5), mainly due to NBD inhibiting ethylene action (Fig. 5). The stimulating effects of NO and KAR1 are dependent on ethylene signaling, even when both are inhibited by 2,5-NBD ethylene inhibitor (Fig. 5). Our findings are in agreement with [54], who found that NBD inhibited the ethylene production in apple seeds, germination of barley and sunflower seeds [55, 56]. In study, we found that ethylene strengthened the influence of KAR1 and NO pre-treatment during the regulation of *Brassica oleracea* seed germination. Our study proves that both molecules, KAR1 and NO, regulate seed germination in an ethylene-dependent manner (Fig. 5). Experiments with NBD suggest that ethylene is necessary for *Brassica* seed germination.

Effect of NO and KAR1 on ACS, ACO and ethylene related genes.

In this study, ACS activity was relatively low during the germination of *Brassica oleracea* seeds after 12 h under KAR1 and NO applications (Fig. 6a). KAR1 and NO induced the fast increase of ACS and ACO activities after 24 and 36 h of incubation; after 48 h, ACS activity declined but ACO activity increased (Fig. 6a and b). However, our experiment suggests that even low activities of ACS and ACO can stimulate dormancy removal. Previous study [17] also found that seed dormancy may be the outcome of low ACC concentration in seeds due to insufficient ACS activity. The pre-treatment of *Brassica* seeds with NO and KAR1 increased the activity of the ACO enzyme (Fig. 6b). In *Pisum sativum* seed germination*,* ethylene regulated the ACO activity [57]. Our results show that during the initial stages of *Brassica* seed germination, the conversion of ACC into ethylene may be independent of ACO. In *Arabidopsis thaliana, Pisum sativum, Lepidium sativum,* and beechnut seeds, the relationship between ACO activity and ACO transcripts, proposed regulation at a transcriptional level during alleviation of dormancy [25, 26, 57].

It has been speculated that ethylene production level, and ACS and ACO gene coding enzymes could be involved in ethylene biosynthesis and seed germination, and our results seem to support this hypothesis (Fig. 6). Previous studies indicated that ethylene could regulate its own synthesis through ACO regulation [13, 44]. Similar mechanisms can also be observed in citrus leaves or in the epicotyls of pea seedlings [58].

In the formation of the ethylene precursor (ACC), ACS genes are dependent on pyridoxal phosphate (PLP). In our study, we selected seven ACS genes and one ACO gene, and found that their expression levels correlated with ethylene production (Fig. 7). Genes with high expression level related with high ethylene production. The ACO genes are responsible for conversion of ACC to ethylene. The expression of ACO and ACS genes was not the same under NO and KAR1 applications. The expressions of *BOACS1, BOACS3, BOACS5, BOACS9* and *BOACS11* were up-regulated, and *BOACS4* and *BOACS7* were down-regulated to a different extent during incubation in water (Fig. 7a, b and c). ACO and ACS genes up-regulation modulated ethylene production under NO and KAR1 applications (Fig. 8).

Previous studies also found different types of ethylene receptors (ETR) in plants [59]. In this study, we used two different types of ethylene receptor genes (*BOETR1* and *BOETR2*) and found that these two genes were different in their expressions and function (Figs. 7 and 9). *BOERT2*’s expression level was higher than that of *BOETR1* under NO and KAR1 treatments. In transgenic tomato plants, the ethylene response is determined by the rate of receptor expressions [60]. Previously [48] found that ethylene receptors that were not bound to ethylene were considered negative regulators because they inhibited the ethylene signal transduction pathway. Ethylene production did not increase significantly during incubation in water (Fig. 5a), suggesting that any ethylene-dependent process might be inhibited during seed dormancy alleviation. In *Brassica* dormant seeds, both ethylene receptor genes *BOETR1* and *BOETR2* were up-regulated during water incubation (Fig. 7b, c). The low sensitivities of *BOETR1* and *BOETR2* to ethylene might be caused by their up-regulation and during incubation in NO and KAR1, both of these receptors exhibited up-regulation. These findings are similar to previous reported findings, proposing the stimulatory influence of NO and KAR1 and the ethylene signaling pathway on seed dormancy release [10, 11, 61, 62]. Some similarities have been detected between NO and KAR1, such as both molecules acting via ethylene in breaking dormancy. In conclusion, we have successfully alleviated nearly 98% of the seed dormancy of *Brassica olereace* after 7 days of NO and KAR1 treatments that modified the ethylene pathway and antioxidant enzyme activity.

## Conclusion

Imbalances between antioxidant enzymes, hydrogen peroxide, superoxide anion, and low levels of ethylene lead to seed dormancy in plants. NO (5 mM) and KAR1 (3 × 10^− 9^ M) treatments alleviates the seed dormancy of *Brassica oleracea* seeds and enhances the hydrogen peroxide, superoxide anion, GA contents, antioxidant enzyme, and ethylene levels required to induce seed germination. Ethylene modulation seems to be an important factor in seed germination induction. However, further studies are required to fully reveal the pathway components, determine the hierarchy of these two compounds, and identification of responsible target genes that would help elucidate the precise roles of NO, KAR1, and ethylene in the secondary dormant *Brassica oleracea* germination process.

## Material and methods

### Plant material, growth conditions and determination of water contents

Secondary dormant *Brassica oleracea* L. seeds were collected from the rapeseed cultivation and breeding lab of Anhui Agricultural University, China. Seeds (25 seeds each in three replicates per treatment) were imbibed in water in the presence of infrared light (100 μmol*·*m^2^*·*s^*−* 1^ radiation) at 5 °C under osmotic potential of − 1600 kPa for 4 days to induce secondary dormancy. After 4 days seeds were stored at − 20 °C. Seeds were sterilized by washing with 3% sodium hypochlorite (Aladin, Shanghai, China), then incubated in distilled water for 8 h, followed by imbibition on pre-wetted muslin cloth for 1 day. The imbibed seeds were cultured in growth chambers under 16 h light and 8 h dark cycle conditions at 21 °C and 70% relative humidity.

To check seed germination [1]; filter paper placed in the petri dishes and moistened with (a) 1.5 ml of distilled water, or (b) KAR1 (3 × 10^− 9^ M) or (c) NO (5 mM) and [2] seeds were placed on wetted filter paper and covered with a transparent lid for 5 days. After every 24 h, germination percentage was recorded up to 5 days. Seeds with visible white radicle (1 mm, after the seed coat was broken) were recorded as germinated. To protect germination from light effect or damage, seeds were grown in growth chamber under a green, safe light at 0.5 μmol m^− 2^ s^− 1^. In order to determine the water contents of treated and non-treated seeds (25 seeds each in three replicates per treatment), seeds were placed on a roller (at 10 °C for 24 h). Water contents were examined by oven-drying the seeds for 5, 10, 20, 30, 40 and 50 h, at 105 °C.

### KAR1 synthesis and treatment of *Brassica* seeds with NO or KAR1 or ACC

KAR1 was prepared by the direct use of methyl pyruvate, dihydro-2H-pyran-3-(4H)-one and Ti-cross aldol [63]. To get the crystal compound, silica gel column chromatography was used to purify the obtained product. HRMS and 1H NMR analysis were performed to confirm the structure. NMR was analyzed (using CD13 as a solvent) on a Bruker AC 200 spectrometer with 200.13MHZ frequency. HRMS was analyzed on a Finnigan MAT 95 spectrometer.

The influence of nitric oxide in light on seed germination was examined following [14] method. HCL (0.1 M) and sodium nitrite (20 mM) were used to prepare acidified nitrite, as recommended previously [11]. The seeds (25 seeds each in three replicates per treatment) were washed using distilled water, following this, seeds were placed on filter paper (one layer) in petri dishes and moistened either with; (**a**) 1.5 ml of distilled water, or (**b**) KAR1 (3 × 10^− 9^ M) with or without1-aminocyclopropane-1-carboxylic acid (ACC 1 × 10^− 4^ M), or (**c**) NO (5 mM) with or without1-aminocyclopropane-1-carboxylic acid (ACC 1 × 10^− 4^ M), or (**d**) 1-aminocyclopropane-1-carboxylic acid (ACC 1 × 10^− 4^ M).

### Hydrogen peroxide (H_2_O_2_) and superoxide anion (O_2_˙ˉ) concentration measurement

Previous methodology [64] was used to measure the concentration level of H_2_O_2_ in seeds. Seeds (25 seeds each in three replicates per treatment) were homogenized in ice with 3 ml cold TCA 0.1% (w/v). The obtained mixture was centrifuged for 15 min at 4 °C at 13,000 RPM. Then (0.5 ml) supernatant and 1 ml freshly prepared KI (1 M) were added to 0.5 ml potassium phosphate buffer (10 mM and pH 7.0). H_2_O_2_ absorbance was measured using a spectrophotometer (Shanghai Yoke Instrument, Shanghai, China) at 390 nm.

Previous methodology [65] was followed to measure the concentration levels of superoxide anion in secondary dormant *Brassica oleracea* seeds. Seeds (25 seeds each in three replicates per treatment) were homogenized on ice with 3 ml cold TCA 0.1% (w/v). The obtained mixture was centrifuged at 13,000 RPM at 4 °C for 15 min. Subsequently, 1.5 ml supernatant and 1.5 ml hydroxylamine hydrochloride (1 mM) were mixed in 50 mM potassium buffer (pH 7.8) in the absence of light and incubated for 30 min at 21 °C. 0.5 ml of the obtained mixture was then added to a mixture of 0.5 ml 2-naphthylamine (7 mM) and 0.5 ml sulphanilamide (17 mM) (in potassium buffer) and incubated for 30 min in the dark at 21 °C. The resultant mixture was again centrifuged for 10 min at 13,000 RPM. The absorbance of the superoxide anion was determined at A540 nm using a spectrophotometer (Shanghai Yoke Instrument, Shanghai, China). Sodium nitrite (NaNO_2_) was used to prepare the calibration curve.

### Analysis of antioxidant enzyme (SOD, CAT, and GR) measurement

EDTA (1 mM), 2% PVP, PMSF (0.01 mM), and DTT (5 mM) were used to ground the secondary dormant seeds (25 seeds each in three replicates per treatment) on ice.

Then resultant samples were added in 5 ml potassium phosphate buffer (0.1 M and pH 7.0) and centrifuged for 15 min at 10,000 RPM at 4 °C. Then obtained mixture desalted in the presence of phosphate buffer using a Sephadex G-250 column (BioRad).

HCl (40 mM), glycine buffer (0.05 M and pH 9.7) and 50 μl epinephrine solution (120 mM) were mixed together to a prepare reaction mixture, as previously described [66, 67] method. Samples (50 μl) were homogenized in reaction mixture and acidified epinephrine was added to start reaction. A spectrophotometer (Shanghai Yoke Instrument, Shanghai, China) monitored the oxidation of epinephrine to adrenochrome at 480 nm.

Aebi’s [68] methodology was followed to measure the catalase (CAT) activity in secondary dormant seeds (25 seeds each in of three replicates per treatment). Samples (50 μl) were homogenized in potassium phosphate buffer (0.07 M) and 6% H_2_O_2_ was added to start reaction. CAT activity was examined using a spectrophotometer (Shanghai Yoke Instrument, Shanghai, China) at 240 nm.

Glutathione reductase **(**GR) activity was measured using [69] method. Twenty-five seeds each in three replicates per treatment were used to measure GR activity. GSSG (5 mM) and potassium phosphate buffer (0.05 M) were mixed together and samples were added in obtained mixture at 21 °C for 10 min. β-NADPH (2 mM) was used to start reaction. GR activity was examined using a spectrophotometer (Shanghai Youke Instrument, Shanghai, China) at 340 nm. Bradford’s [70] methodology was used to measure the protein content. Bovine serum albumin (BSA) was used as a standard.

### Determination of ABA and GA contents

ABA contents were measured according to [71] method. In presence of liquid nitrogen seeds (25 seeds in each of the three replicates per treatment) were ground into powder. Then samples were homogenized in methanol. D6-ABA was applied as an internal standard. To purify the samples, formic acid (7%) was added in methanol in the presence of Oasis Max solid phase. In next step samples were injected into chromatography–tandem mass spectrometry system, equipped with a triple quadruple tandem mass spectrometer and ultra-performance liquid chromatograph.

Determination of GA contents was performed as described by [71] method. In the presence of liquid nitrogen, seeds (25 seeds in each of the three replicates per treatment) were ground into powder. Methanol (85% v/v) was used for extraction. The resultant mixture was purified by ethyl ether extraction, solid-phase extraction and reversed-phase. For quantitative analysis, samples were injected into capillary electrophoresis-mass spectrometry (CE-MS).

### Measurement of ethylene production and treatment with NBD

Secondary dormant seeds (25 in each of the three replicates per treatment) were treated with (a) NO (5 mM), (b) KAR1 (3 × 10^− 9^ M), and (c) ACC (1 × 10^− 4^ M) or without ACC for 12, 24, 36, and 48 h, as described previously [44]. In the presence of moistened filter paper, glass containers (10 ml) were used to incubate the treated seeds in the dark at 21 °C for 3 h. A syringe was used to take the sample from glass container. Following that, sample (1 ml) was injected into a Hewlett–Packard 5980 GC equipped with FID, a Pro pack Q 80/100 mesh and stainless steel column. Then samples were placed in an oven at 60 °C for isothermal separation. The measuring unit for ethylene is pl h^− 1^ 25 seed^− 1^.

NBD, an inhibitor of ethylene, was applied to check the effect of ethylene on secondary seed germination. In tightly closed glass containers (500 ml), secondary dormant seeds (25 in each of the three replicates per treatment) were placed on filter paper and moistened with; (**a**) KAR1 (3 × 10^− 9^ M), or (**b**) NO (5 mM) in the presence of 2,5-norbornadiene (NBD), according to [44] method. NBD (3 × 10^− 5^ and 6 × 10^− 5^ M) in liquid form was applied under the lid of the container. After 5 h of treatment, seeds were rinsed three times with deionized water and positioned for germination assay.

### Determination of ACC contents, ACC synthase and ACC oxidase activities

ACC contents were determined using [72] method. Ethanol (2 ml and 80% v/v) was used to homogenized treated (with NO and KAR1) and non-treated seeds (25 seeds in each of the three replicates per treatment). To remove the ethanol, samples were centrifuged for 15 min at 13,000 RPM at 4 °C. Following this, the ACC contents were measured at room temperature using a Hewlett–Packard 5980 GC. ACC content was calculated in μmol g − 1 FW.

ACC synthase (ACS) activity was examined using the [72, 73] method. Dithiothreitol (DTT) 5 mM, polyvinylpolypyrrolidone (PVPP) 2% (w/v), phenylmethanesulfonyl fluoride (PMSF) 0.01 mM, HEPES– KOH buffer (0.1 M pH 8.5) and 10 μM pyridoxal phosphate (PLP) were mixed together to prepare a homogenize buffer. Seeds were homogenized in homogenize buffer on ice.

Samples were vortexed for 10 s and then centrifuged at 4 °C for 15 min at 13,000 RPM.

To prepare the reaction mixture, PLP (10 μM), HEPES– KOH (0.1 M and pH 8) and S-adenosyl methionine (200 μM) were mixed together. Then samples were incubated (at 37 °C) in a glass container in the presence of reaction mixture (0.6 ml) for 60 min. HgCl_2_ 0.2 ml (10 mM) was used to stop reaction.

ACC oxidase in seeds was measured according to the [74] protocol. First, extraction buffer (E.B) was prepared by mixing glycerol 10% (v/v), 5% (v/v) Triton X-100, Tris–HCl 0.1 M, 7 mM DTT, 25 mM Na-ascorbate and 0.15 mM PMSF and then samples were homogenized in 4 ml E.B. ACC 2 mM, NaHCO_3_ 35 mM, DTT 1 mM, FeSO4 25 μM, Na-ascorbate 30 mM, NaHCO_3_ 30 mM and Tris–HCl 0.1 M were used in the preparation incubation buffer. Samples were centrifuged at 4 °C for 15 min at 13,000 RPM, and the resultant mixture was added to incubation buffer. The obtained mixture (for 1 h) was placed on a shaking machine at 37 °C. ACO activity was calculated in pl h^− 1^ mg^− 1^ protein.

### RNA extraction and RT-qPCR of ethylene related genes

To extract the RNA of secondary dormant *Brassica oleracea* seeds, seeds were pre-treated with NO (5 mM) and KAR1 (3 × 10^− 9^ M) for 0, 12, 24, 36, and 48 h to observe the expression levels of ethylene related genes [49]. In this study, we selected 10 ethylene-related genes (Table 1). Twenty-five seeds (in each of the three replicates) were treated with (**a**) NO (5 mM), or (**b**) KAR1 (3 × 10^− 9^ M), or (**c**) water. To prepare the first-strand cDNA, DNase I was added to 1 μg aliquots of total RNA using an Omega Bio-Tek kit (Biotechnology, Shanghai, China). Nuclease-free water was used for dilution (10%) of obtained samples. Following this, SYBR Select master mix (Biotechnology, Shanghai, China) was used to perform RT-qPCR. Reactions were started at 95 °C (10 min) followed by 50 cycles amplification at 95 °C (10 s), 60 °C (20 s), and 72 °C (20 s), and melting at 95 °C (for 2:30 min, 60 °C, 30 s), then continuously increased to 95 °C. Genes and qPCR primer sequences are given in Additional file 1: Table S1.

**Table 1:** List of primer sequences used in the experiments

Gene Name	NCBI Reference Number	Direction	Primer Sequences (5’…..3’)
BO-ACS1	X82273	Forward	GCAGAGAAGCAAGACCAGAA
		Reverse	TTTCTCGCCGTGTCCGTC
BO-ACS3	AF338652	Forward	GGATAGTGATGAGTGGCGG
		Reverse	TCGGCGAGGCAGAACATA
BO-ACS4	AB086353	Forward	GGATAGTGATGAGTGGCGG
		Reverse	CCCCGCCACTCATCACTA
BO-ACS5	AF074930	Forward	TGAAAACCAGCTATGTTTCGATCTT
		Reverse	AAGATCGAAACATAGCTGGTTTTCA
BO-ACS7	AF338651	Forward	CAAATGGGGCAAGCGGAGAATCAGG
		Reverse	CCTGATTCTCCGCTTGCCCCATTTG
BO-ACS9	AF074929	Forward	TGCTTTTCTTTTACCCACTC
		Reverse	GCTCCCGTTCTCCATTTC
BO-ACS11	AF074928	Forward	AACAAACTACTATGTAAAAAATCCTG
		Reverse	AACTGATTCTTCGTTTTTTTTC
BO-ACO1	X81628	Forward	GAGAAGTTGAGGATGTTGATTG
		Reverse	CCAATCAACATCCTCAACTTC
BO-ETR1	AF047476	Forward	GCTCAAACACAGTCTTTAGCGAC
		Reverse	ATCACACTAAACCTCGCACCAG
BO-ETR2	AB078598	Forward	GGTGATAACCAACGGCAGG
		Reverse	CGTGGCTCCTTAGGCTGAA
BO-ACT1	AF044573	Forward	GCTCCCAGGGCTGTTTTC
		Reverse	CATCAGCCTCAGCCATTTTT

### Statistical analysis

Data presented in all graphs were mean ± standard errors (SEs) using two-way analysis of variance (ANOVA). Multiple comparisons were made using Tukey’s test. *P* ≤ 0.05 was used to measure the significant differences between the indicated treatment groups and control.

## Supplementary information

**Additional file 1: Table S1.** List of primer sequences used in the experiments.

## Data Availability

Sequence data from RNA-seq described in this article had been released at NCBI (https://www.ncbi.nlm.nih.gov/).The detailed information of genes and primers is listed in Additional file 1: Table S1.

## Abbreviations

ACC: Synthase2,5-norbornadiene (NBD)
ACO: Aminocyclopropane-1-carboxylic acid ACC oxidase
CAT: Catalase
ETR: Ethylene receptors
GR: Glutathione reductase
H_2_O_2_: Hydrogen peroxide
KAR1: Karrikin1
NO: Nitric oxide
O_2_˙ˉ: Superoxide anion
SOD: Superoxide dismutase
