# Supplementary material for: Alleviating dormancy in Brassica oleracea seeds using NO and KAR1 with ethylene biosynthetic pathway, ROS and antioxidant enzymes modifications
Source: BMC Plant Biol. 2019 Dec 23;19:577. doi: 10.1186/s12870-019-2118-y (PMC6929364; doi:10.1186/s12870-019-2118-y)
Supplement: Supplementary file 1 — Additional file 1: Table S1. List of primer sequences used in the experiments. [file 12870_2019_2118_MOESM1_ESM.docx]

| **Gene Name** | **NCBI Reference Number** | **Direction** | **Primer Sequences (5’…..3’)** |
| --- | --- | --- | --- |
| **BO-ACS1** | X82273 | Forward | GCAGAGAAGCAAGACCAGAA |
|  |  | Reverse | TTTCTCGCCGTGTCCGTC |
| **BO-ACS3** | AF338652 | Forward | GGATAGTGATGAGTGGCGG |
|  |  | Reverse | TCGGCGAGGCAGAACATA |
| **BO-ACS4** | AB086353 | Forward | GGATAGTGATGAGTGGCGG |
|  |  | Reverse | CCCCGCCACTCATCACTA |
| **BO-ACS5** | AF074930 | Forward | TGAAAACCAGCTATGTTTCGATCTT |
|  |  | Reverse | AAGATCGAAACATAGCTGGTTTTCA |
| **BO-ACS7** | AF338651 | Forward | CAAATGGGGCAAGCGGAGAATCAGG |
|  |  | Reverse | CCTGATTCTCCGCTTGCCCCATTTG |
| **BO-ACS9** | AF074929 | Forward | TGCTTTTCTTTTACCCACTC |
|  |  | Reverse | GCTCCCGTTCTCCATTTC |
| **BO-ACS11** | AF074928 | Forward | AACAAACTACTATGTAAAAAATCCTG |
|  |  | Reverse | AACTGATTCTTCGTTTTTTTTC |
| **BO-ACO1** | X81628 | Forward | GAGAAGTTGAGGATGTTGATTG |
|  |  | Reverse | CCAATCAACATCCTCAACTTC |
| **BO-ETR1** | AF047476 | Forward | GCTCAAACACAGTCTTTAGCGAC |
|  |  | Reverse | ATCACACTAAACCTCGCACCAG |
| **BO-ETR2** | AB078598 | Forward | GGTGATAACCAACGGCAGG |
|  |  | Reverse | CGTGGCTCCTTAGGCTGAA |
| **BO-ACT1** | AF044573 | Forward | GCTCCCAGGGCTGTTTTC |
|  |  | Reverse | CATCAGCCTCAGCCATTTTT |

**Table S1. List of primer sequences used in the experiments.**
